# Spermidine Affects Cardiac Function in Heart Failure Mice by Influencing the Gut Microbiota and Cardiac Galectin-3

**DOI:** 10.3389/fcvm.2021.765591

**Published:** 2021-12-02

**Authors:** Yufeng Chen, Zhiqin Guo, Shaonan Li, Zhen Liu, Pingan Chen

**Affiliations:** ^1^Department of Cardiology, The Second Affiliated Hospital, School of Medicine, South China University of Technology, Guangzhou, China; ^2^Department of Cardiology, Guangzhou First People's Hospital, Guangzhou, China

**Keywords:** heart failure, spermidine, microbiota, cardiac fibrosis, galectin-3

## Abstract

Spermidine, which can be synthesized by the gut microbiota, can prevent cardiac hypertrophy and delay the progression to heart failure (HF). However, it is not clear whether the effect of spermidine on cardiac function is mediated by modulating the gut microbiota when HF occurs. Female HF Kunming mice induced by transverse aortic constriction were administered spermidine (HF+S group) or its antagonist (HF+SR group). Echocardiography, messenger ribonucleic acid (RNA) and protein expression of galectin-3 in the heart, cardiomyocyte apoptosis assays and gut microbiota analysis were detected. Left ventricular end-diastolic volume and diameter (LVVd and LVDd), and left ventricular end-systolic volume and diameter in the HF+SR group were significantly enlarged compared with those in the HF group (all *P* < 0.05). The HF+S group had a smaller LVDd and LVVd than the HF+SR group (5.01 ± 0.67 vs. 6.13 ± 0.45 mm, *P* = 0.033; 121.44 ± 38.74 vs. 189.94 ± 31.42 μL, *P* = 0.033). The messenger RNA and protein expression of galectin-3 and the number of apoptotic cardiomyocytes increased significantly in the HF+SR group compared to the HF group. Gut microbiota analysis showed that spermidine antagonists reduced the *Firmicutes/Bacteroidetes* ratio and changed the microbial community richness and diversity. In conclusion, spermidine can improve cardiac function in HF, and the regulation of gut microbiota and cardiac fibrosis may be a factor in the effect of spermidine on the improvement of cardiac function.

## Introduction

Cardiac function and gut microbiota can affect each other. Reduced cardiac output causes intestinal wall ischemia, edema and structural disruption of the intestinal epithelial barrier function in heart failure (HF), leading to increased intestinal permeability, which in turn, contributes to the progression of HF (1). Furthermore, the impairment of intestinal barrier function leads to the translocation of gut bacterial deoxyribonucleic acid (DNA) and/or endotoxins into the bloodstream (2) and contributes to the deterioration of cardiac function (3). Studies have shown that in chronic HF, the gut microbiota is characterized by large compositional shifts with low bacterial richness and depletion of the core microbiota (4, 5). Therefore, the intestinal microbiota plays an important role in the development and progression of HF (6–8).

Spermidine is a natural polyamine present in all living organisms at levels from micromolar to millimolar that is critically involved in the maintenance of cellular homeostasis (9, 10), usually with endogenous spermidine concentrations decreasing during the natural process of organismal aging (11, 12). Some studies have shown that dietary spermidine protects against cardiovascular aging (13), prevents cardiac hypertrophy and delayed the progression to HF (14), suggesting a beneficial effect on HF. One possible mechanism is that spermidine can enhance cardiac autophagy, mitophagy and mitochondrial respiration (14, 15). Moreover, the gut microbiota is significantly related to the synthesis of spermidine (16) and some gut microorganisms contain spermidine synthase (17). Spermidine can also enhance the gut barrier integrity and alter the gut microbiota in diet-induced obese mice (18). Although spermidine and the gut microbiota are both associated with HF, it is not clear whether cardiac function can be affected by spermidine through modulating the gut microbiota when HF occurs. We speculated that regulation of the gut microbiota might be a contributing factor to the effect of spermidine on the improvement of cardiac function. A close relationship may exist among spermidine, HF and the gut microbiota, and the cardioprotective role of spermidine, especially to HF, may be mediated by optimizing the gut microbiota composition.

In this study, we investigated the effect of spermidine on HF by altering its levels and assessed the association between spermidine and gut microbiota in an HF model induced by transverse aortic constriction (TAC) to explore whether spermidine can affect cardiac function by modulating the gut microbiota.

## Materials and Methods

### Aortic Constriction

TAC was performed as described previously (19). Briefly, female Kunming mice, weighing 45–52 g, 11 weeks old, after anesthetization with pentobarbital sodium (40 mg/kg, intraperitoneally injected), were intubated and ventilated with a small animal respirator, at a rate of 130 breaths/min and a tidal volume of 0.4 ml. Aortic constriction was applied by tying a 7.0 silk string ligature around a 27-gauge needle and then removing the needle. Sham-operated mice served as controls and were subjected to the same surgeries except for the ligation of the aorta. These experiments conformed to the Guide for the Care and Use of Laboratory Animals published by the US National Institutes of Health (NIH publication No. 85-23, revised 2011). The protocol was approved by the institutional ethics committee of Guangzhou First People's Hospital.

**Figure 1:**
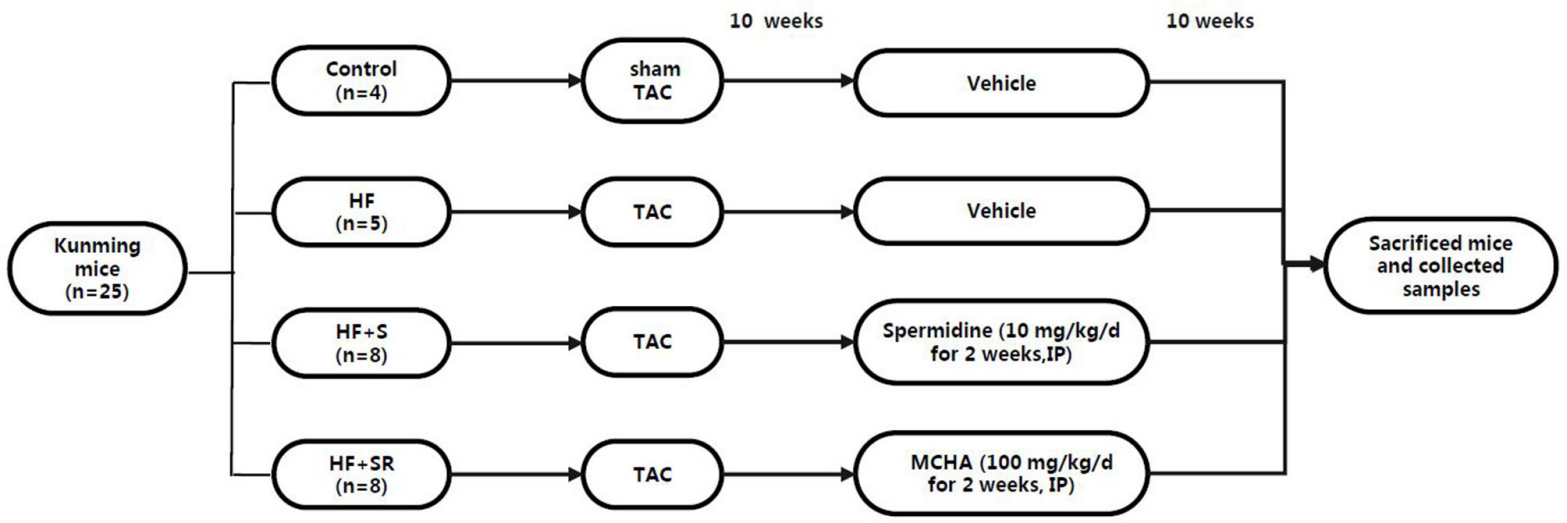
The animal grouping and time line of the experimental protocol.

The HF mice were then randomly divided into three groups at 70 days post-operation: the HF group (*n* = 5), treated with saline; the HF+S group (*n* = 8), administered with spermidine by intraperitoneal injection of 10 mg/kg/d (Sigma-Aldrich, USA, diluted with saline solution) for 7 days (20); and the HF+SR group (*n* = 8), treated with trans-4-methylcycloh exylamine (4-MCHA), an antagonist of spermidine, by intraperitoneal injection of 100 mg/kg/d (Sigma-Aldrich, USA, diluted with saline solution) for 7 days (21). All animals had free access to common food and water. At 70 days after administration, the mice were sacrificed by pentobarbital sodium overdose (150 mg/kg), and then fecal samples and left ventricular tissues were harvested for the analysis (Figure 1).

### Quantitative Real-Time Polymerase Chain Reaction

Total ribonucleic acid (RNA) was isolated from left ventricular biopsies using TRIzol reagent (Invitrogen) according to the manufacturer's protocol. Primers were designed to detect galectin-3 gene expression (forward: GAGTACTAGAAGCGGCCGAG, reverse: CTGTGCCGCTCACCTGATTA) based on the sequences available in NCBI database (at http://ncbi.nlm.nih.gov) using Primer software. After measuring RNA concentration, 1.5 μg RNA was treated with DNase I (Invitrogen) and used for cDNA synthesis by reverse transcriptase M-MLV (Takara, Japan). The galectin-3 messenger RNA (mRNA) levels were measured with CFX96 quantitative real-time polymerase chain reaction (QT-PCR) system (Bio-Rad). The relative amounts of mRNA were determined based on 2-ΔΔCt calculations.

### Echocardiography

Transthoracic echocardiography was performed using a Vevo 2100 imaging system equipped with a 15–30 MHz linear array transducer (VisualSonics, Inc., Canada) by a single blinded observer as described previously (14). The following parameters were measured and averaged from 3 cardiac cycles: stroke volume, left ventricular end-diastolic volume and diameter (LVVd and LVDd), and left ventricular end-systolic volume and diameter (LVVs and LVDs), left ventricular anterior and posterior wall thickness in systole and diastole (LVAWs, LVAWd, LVPWs, and LVPWd). The left ventricular mass was calculated following the previously described method of Gao et al.: left ventricular mass = [(LVDd + LVAWd + LVPWd)^3^ – LVDd ^3^] × 1.055, where 1.055 is the gravity of the myocardium (22). Fractional shortening (FS, %) was calculated using the equation: 100 × [(LVDd – LVDs)/LVDd]. Left ventricular ejection fraction (LVEF, %) was calculated as 100 × (LVVd – LVVs)/LVVd.

### Western Blot Analysis

Left ventricular tissues were homogenized in RIPA buffer (Beyotime Biotechnology, China) to obtain whole cell lysates and centrifuged to isolate the protein. Fifty micrograms of protein was subjected to SDS-polyacrylamide gel electrophoresis and blotted onto a PVDF membrane. After blocking with 5% non-fat milk, the membranes were incubated with the following primary antibodies: galectin-3 (Abcam, Shanghai, China), spermidine (Abcam, Shanghai, China) or β-actin (Proteintech, Wuhan, China). Then, after incubation with anti-rabbit HRP-conjugated IgG (1:2,500, Boster, China) or anti-mouse HRP-conjugated IgG (1:2,500, Boster, China) for 1 h at room temperature, the immunoreactive bands were visualized by chemiluminescence reagents (ECL; KeyGEN BioTECH, Nanjing, China). The band intensity was quantified using ImageJ analysis software in a blinded manner, and all bands were normalized to the corresponding β-actin bands.

### Immunohistochemistry

For galectin-3 and spermidine immunohistochemistry, formalin-fixed left ventricular tissues were embedded in paraffin and sectioned into 5-μm-thick sections. After deparaffinization, rehydration and pre-treatment with hydrogen peroxide, the sections were incubated with primary antibodies against galectin-3 (Abcam, Shanghai, China) and spermidine (Abcam, Shanghai, China) overnight at 4°C before being incubated with a secondary antibody for 1 h at room temperature. The sections were stained with DAB (Servicebio, China) followed by counterstaining with hematoxylin. Three entire sections per heart were examined by skilled observers blinded to the treatment group.

### Terminal Deoxynucleotidyl Transferase–Mediated dUTP Nick-End Labeling Staining

TUNEL assays were performed to detect apoptotic cells in the heart tissue sections according to the manufacturer's protocols (Roche, China). Briefly, the left ventricular tissues were deparaffinized and rehydrated with serial changes in xylene and five different concentrations of ethanol. After proteinase K and endogenous peroxidase treatment, the heart tissue sections were stained with DAB (Servicebio, China). The TUNEL-positive cells were quantified using Image-Pro Plus analysis software.

### Microbiome Sequencing and Analysis

Mouse fecal samples were collected under sterile conditions by pressing on the outer wall of the colon to push its contents into sterile tubes, at which time they were snap frozen in liquid nitrogen and then stored at −80°C until analysis. Total genomic DNA from the samples was extracted using the CTAB/SDS method by Novogene (Tianjin, China). The DNA concentrations and purity were analyzed on 1% agarose gels. The diluted DNA was used as the template to amplify the 16S rRNA (16S V4: 515F-806R) with specific barcoded primers. All polymerase chain reaction (PCR) reactions were carried out with Phusion® High-Fidelity PCR Master Mix (New England Biolabs). The PCR products were purified with the GeneJET Gel Extraction Kit (Thermo Scientific). The sequencing libraries were generated using the NEB Next® UltraTM DNA Library Prep Kit for Illumina (NEB, USA) and index codes were added. The library quality was assessed on the Qubit@ 2.0 Fluorometer (Thermo Scientific) and the Agilent Bioanalyzer 2100 system. Then the qualified library was sequenced on the Illumina MiSeq platform, and 250 bp paired-end reads were generated. The paired-end reads were combined using FLASH (V1.2.7) and assigned to the samples according to their barcodes.

Sequence analysis was conducted by Uparse software (V7.0.1001) and sequences with ≥ 97% similarity were assigned to the same operational taxonomic units (OTUs). Alpha diversity was analyzed through 3 parameters, including Chao 1, abundance coverage estimator (ACE), observed- species and phylogenetic diversity (PD)-whole tree. The linear discriminant analysis (LDA) effect size (LEfSe) was selected for the quantitative analysis of the microbial biomarkers within the different groups. The differences between the groups were assessed according to the LDA scores [OTUs with (log10)]. Cluster assessment was preceded by principal component analysis (PCA) using QIIME software (V1.7.0).

### Statistical Analyses

Continuous variables were expressed as the mean ± standard deviation. Differences among the means were evaluated with a two-independent-sample *t*-test or one-way ANOVA test with S-N-K analysis, as appropriate. *P*-values were two-sided and considered significant when < 0.05. Statistical analyses were carried out using the SPSS version 17.0 software package (SPSS Inc., Chicago, USA).

## Results

### Assessment of the HF Model Induced by Aortic Constriction and Grouping

During the observation period after the operation, three mice died, while all sham-operated animals (controls, *n* = 4) survived. The TAC mice showed some signs of congestive HF such as anorexia, dyspnea and lethargy at 70 days post-operation, indicating the appearance of HF. Echocardiography showed that LVDs, LVDd, LVVs, LVVd, left ventricular mass average weight (LV Mass AW), and LV Mass AW (corrected) were significantly increased, and LVEF and FS were significantly decreased in the HF mice compared to the sham-operated mice (all *P* < 0.05, Table 1).

**Table 1:** Comparisons of echocardiographic parameters between sham-operated and heart failure groups at 10 weeks post-operation and before administration.

	**Heart rate (bpm)**	**LVDs (mm)**	**LVDd (mm)**	**LVVs (uL)**	**LVVd (uL)**	**FS (%)**	**LVEF (%)**	**LV mass AW (mg)**	**LV mass AW corrected (mg)**
Sham (*n* = 4)	438 ± 35	2.54 ± 0.56	4.12 ± 0.54	24.69 ± 13.71	76.63 ± 24.48	38.79 ± 5.32	69.29 ± 7.12	138.03 ± 29.41	110.42 ± 23.53
HF (*n* = 21)	499 ± 40	3.53 ± 0.47	4.89 ± 0.42	53.45 ± 17.04	113.38 ± 23.67	27.89 ± 5.98	53.39 ± 9.12	218.56 ± 46.25	174.84 ± 37.00
*P*	0.008	0.004	0.011	0.012	0.023	0.008	0.010	0.010	0.010

*Values presented as mean ± SD. bpm, beats per minute; LVDs, left ventricular end-systolic diameter; LVDd, left ventricular end-diastolic diameter; LVVs, left ventricular end-systolic volume; LVVd, left ventricular end-diastolic volume; FS, fractional shortening; LVEF, left ventricular ejection fraction; LV Mass AW, left ventricular mass average weight*.

### Variation in Echocardiographic Parameters After the Administration of Spermidine or Its Antagonist

**Figure 2:**
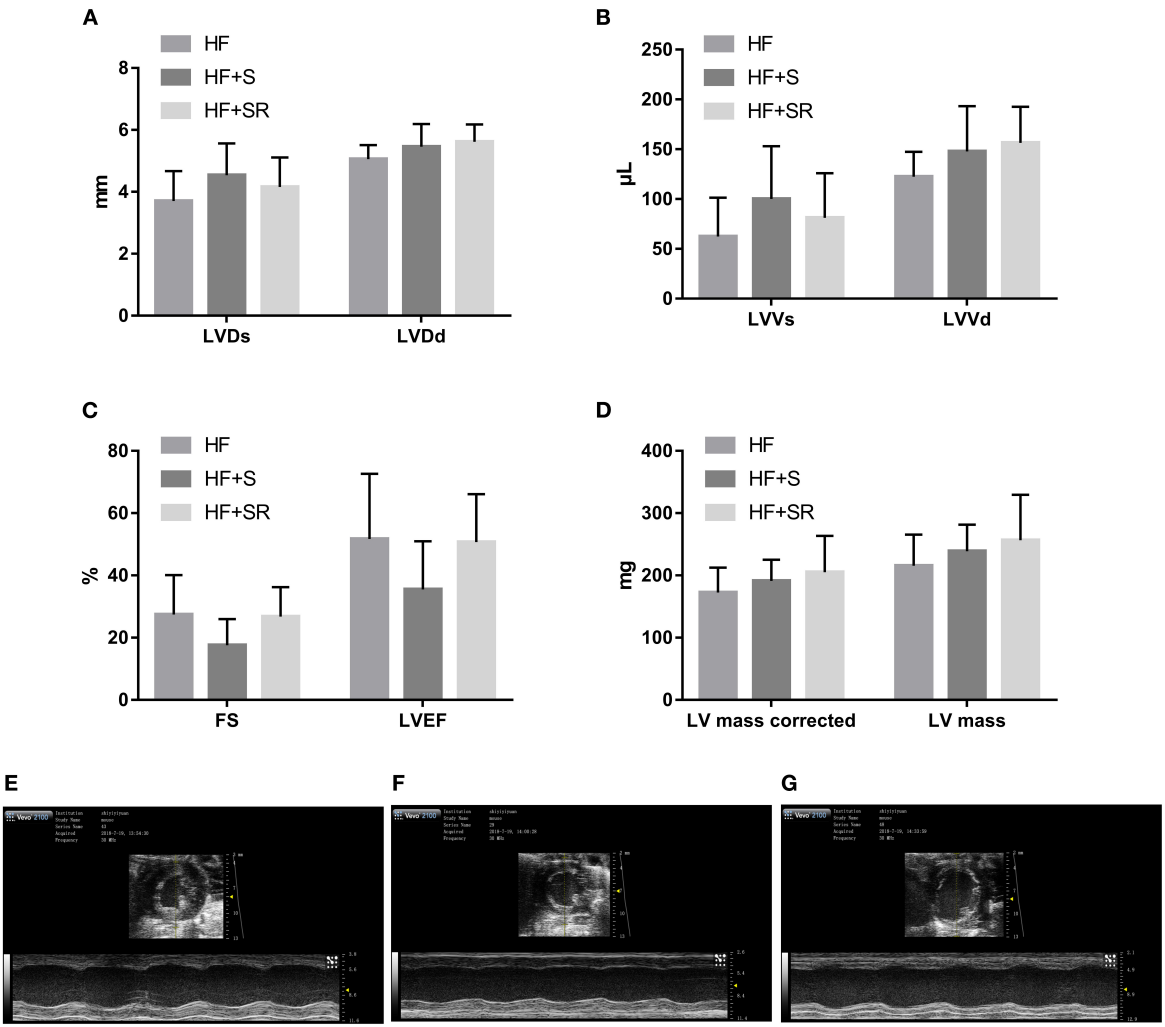
Difference in the echocardiographic parameters among the HF (*n* = 5), HF+S (*n* = 8) and HF+SR (*n* = 8) mice at 14 days after administration. There were no differences in LVDs, LVDd **(A)**, LVVs, LVVd **(B)**, FS, LVEF **(C)**, LV mass or LV mass corrected **(D)** among the three groups. Representative echocardiographic images showing cardiac function and dimensions in the HF **(E)**, HF+S **(F)**, and HF+SR **(G)** mice.

**Figure 3:**
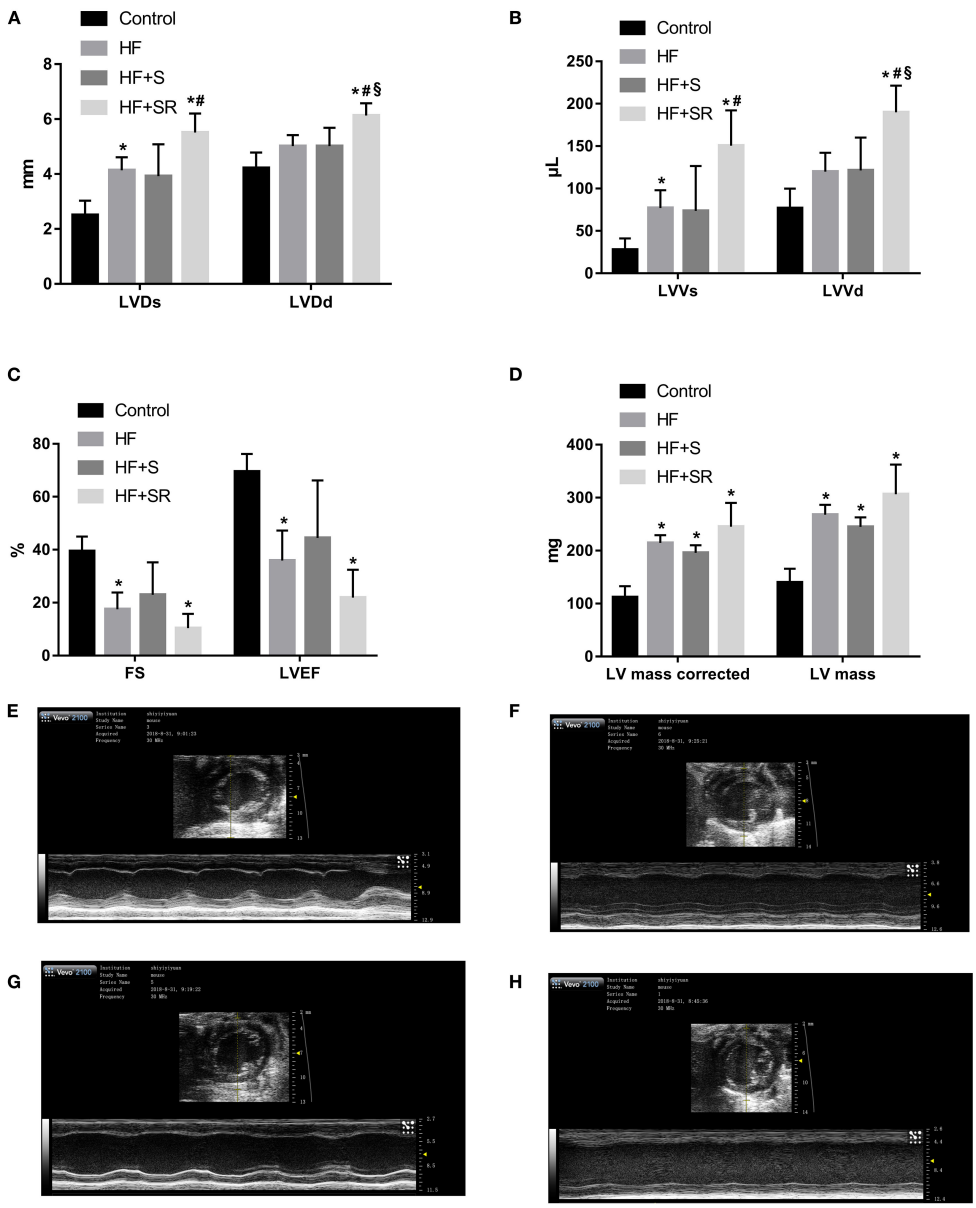
Comparison of echocardiographic parameters among the different groups at 56 days after administration. Comparisons of LVDs, LVDd **(A)**, LVVs, LVVd **(B)**, FS, LVEF **(C)**, LV mass and LV mass corrected **(D)** among the control (*n* = 4), HF (*n* = 5), HF+S (*n* = 8), and HF+SR (*n* = 8) mice. Representative echocardiographic images in the control **(E)**, HF **(F)**, HF+S **(G)**, and HF+SR **(H)** mice. *P < 0.05 vs. controls, ^#^*P* < 0.05 vs. HF group, and ^§^*P* < 0.05 vs. HF+S group.

After administration of spermidine or 4-MCHA, the HF mice were examined by Doppler echocardiography. Figure 2 shows that the echocardiographic parameters, including LVDs, LVDd, LVVs, LVVd, FS, LVEF, LV mass AW, and LV mass AW (corrected) were not significantly different at 14 days after administration among the three groups: the HF, HF+S, and HF+SR groups. However, statistically significant differences were noted 56 days after administration. The LVDs, LVDd, LVVs, and LVVd in the HF+SR group were greatly enlarged (5.51 ± 0.69 vs. 4.14 ± 0.47 mm, *P* = 0.033; 6.13 ± 0.45 vs. 5.02 ± 0.40 mm, *P* = 0.019; 150.44 ± 41.68 vs. 76.92 ± 21.08 μL, *P* = 0.040; 189.94 ± 31.42 vs. 119.83 ± 22.31 μL, *P* = 0.022) compared with those in the HF group. Furthermore, LVDd and LVVd were significantly smaller (5.01 ± 0.67 vs. 6.13 ± 0.45 mm, *P* = 0.033; 121.44 ± 38.74 vs. 189.94 ± 31.42 μL, *P* = 0.033) in the HF+S group than in the HF+SR group (Figure 3).

### The mRNA Expression of Galectin-3 in the Heart

**Figure 4:**
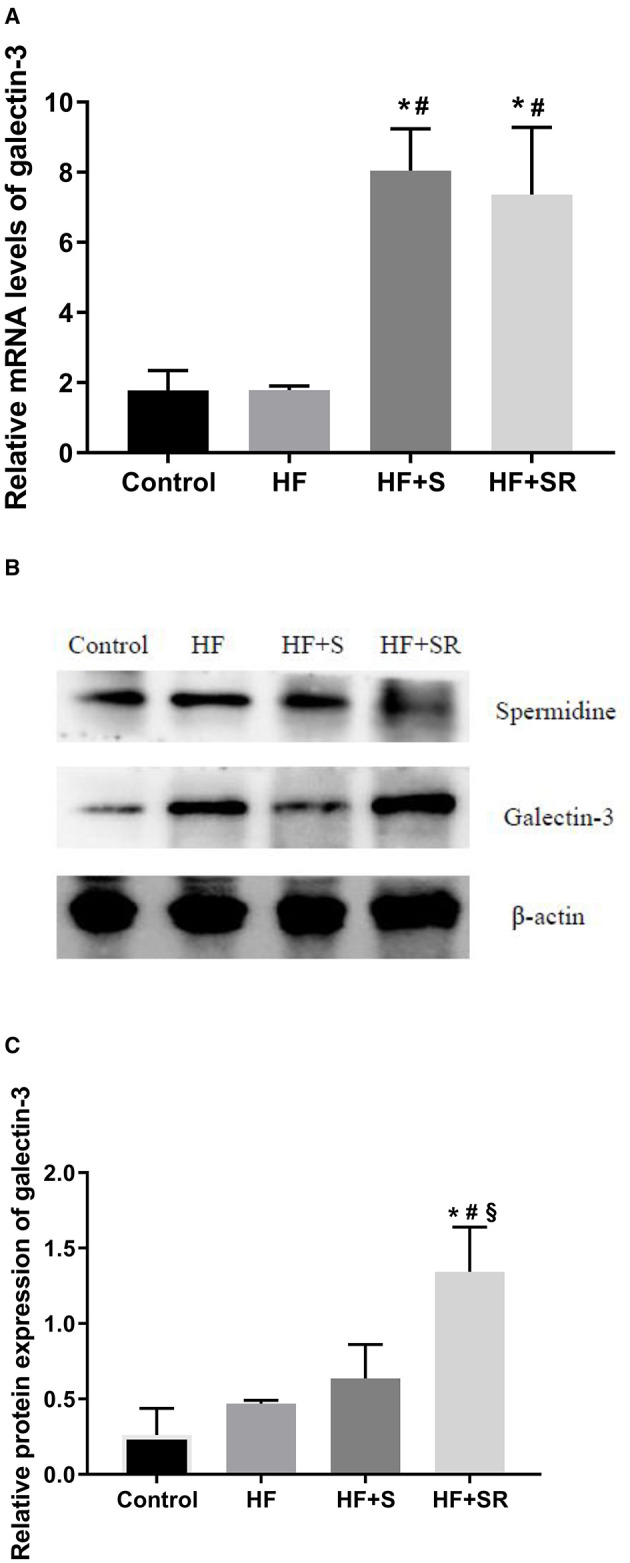
The mRNA and protein expression of galectin-3 in the heart in different groups. Relative mRNA levels of galectin-3 in the heart **(A)**. Representative immunoblots for galectin-3 in the heart **(B)**. Quantitation of galectin-3 protein levels in the heart **(C)**. *P < 0.05 vs. controls (*n* = 4), ^#^*P* < 0.05 vs. HF group (*n* = 5) and ^§^*P* < 0.05 vs. HF+S group (*n* = 8).

Relative gene expression of galectin-3 in the left ventricular tissues was analyzed using QT-PCR at 70 days after administration. The results showed that galectin-3 expression was significantly increased in the two treated groups. The galectin-3 mRNA levels increased 3.5-fold in the HF+S group and 3.1-fold in the HF+SR group compared to the HF group (both *P* < 0.05). However, there was no difference between the HF+S and HF+SR groups (Figure 4A).

### The Protein Expression of Galectin-3 in the Heart

To investigate the protein expression of galectin-3 in the heart, western blotting was conducted. The results showed that the expression levels of spermidine were elevated in the HF+S group, and the relative protein expression levels of galectin-3 were higher in the HF+SR group than in the HF and HF+S groups (~2.1-fold, all *P* < 0.05). However, no difference was observed in the protein expression of galectin-3 between the HF and HF+S groups (Figures 4B,C).

### Immunohistochemistry of Galectin-3 in the Heart Tissue

**Figure 5:**
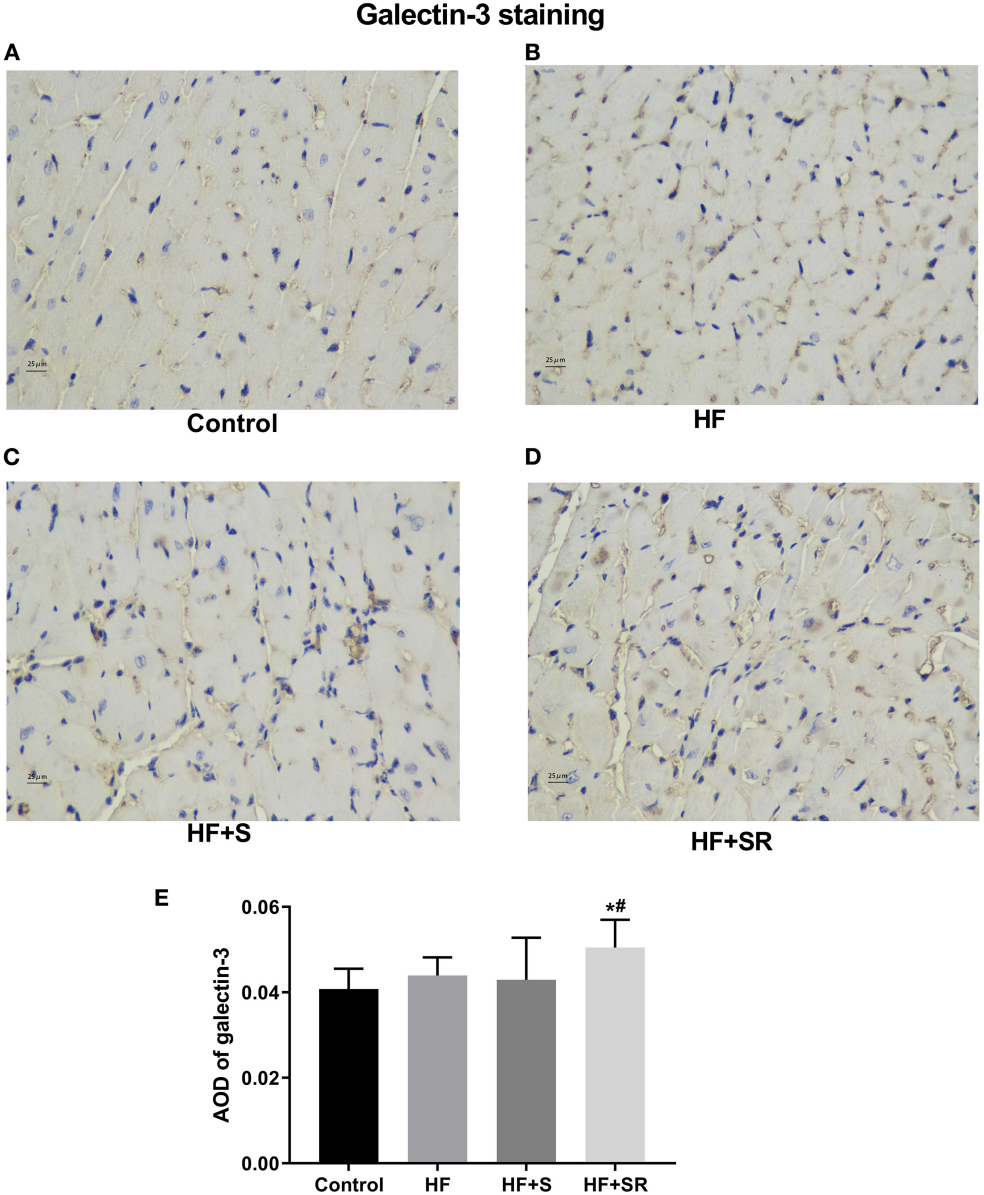
Immunohistochemistry of galectin-3 in heart tissues from different groups. The controls had almost no galectin-3 expression **(A)**. The HF (*n* = 5) and HF+S (*n* = 8) mice had some galectin-3 expression **(B,C)**. The HF+SR mice (*n* = 8) showed significantly stronger staining for galectin-3 **(D)**. Semiquantitative analysis of galectin-3 levels by measuring AOD **(E)**. *P < 0.05 vs. controls, ^#^*P* < 0.05 vs. HF group.

The protein expression of galectin-3 in the heart was also confirmed by immunohistochemistry and semiquantitative analysis, indicated as the values of the average optical density (AOD) measured by Image-Pro Plus 6.0. Immunohistochemistry staining showed that the protein expression of galectin-3 was significantly increased in the HF+SR group compared with the control and HF groups (AOD, 0.051 ± 0.005 vs. 0.040 ± 0.005 and 0.043 ± 0.005, both *P* < 0.05). There was also no difference in the expression of galectin-3 between the HF and HF+S groups (AOD, 0.043 ± 0.005 vs. 0.043 ± 0.010, *P* = 0.976) (Figure 5).

### Effect of Spermidine on Cardiomyocyte Apoptosis

**Figure 6:**
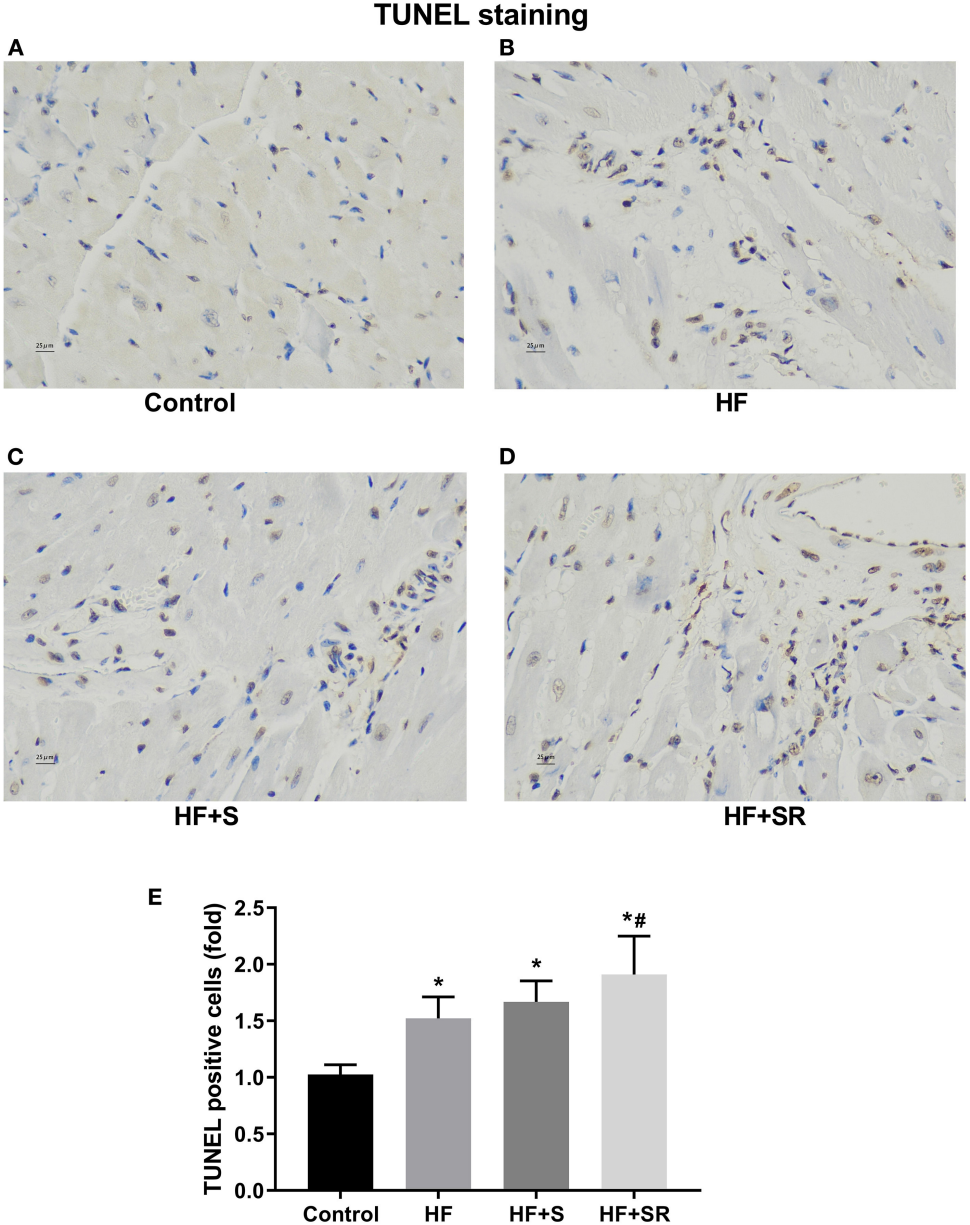
Differences in the number of apoptotic cardiomyocytes among the four groups. Representative photomicrographs of ventricular tissue stained for TUNEL. The controls had very few TUNEL-positive cells **(A)**. Many TUNEL-positive cells appeared in the HF (*n* = 5) and HF+S (*n* = 8) mice **(B,C)**. The percentage of TUNEL-positive cells was significantly increased in the HF+SR mice (*n* = 8) **(D)**. Quantitative analysis of TUNEL-positive cells in the heart sections **(E)**. *P < 0.05 vs. controls, ^#^*P* < 0.05 vs. the HF group.

To assess the effect of spermidine on cardiomyocyte apoptosis, a TUNEL assay was used. Brown-stained cells accompanied by condensed or fragmented nuclei were considered TUNEL-positive cells. The number of apoptotic cardiomyocytes was significantly increased in the HF, HF+S and HF+SR groups compared with the controls. There was a modest 0.5-fold increase in the number of apoptotic cells in the HF+SR group compared to the HF group, but no difference was observed between the HF and HF+S groups (Figure 6).

### Compositional Alteration of Gut Microbiota in Mice With Different Treatment Schemes

**Figure 7:**
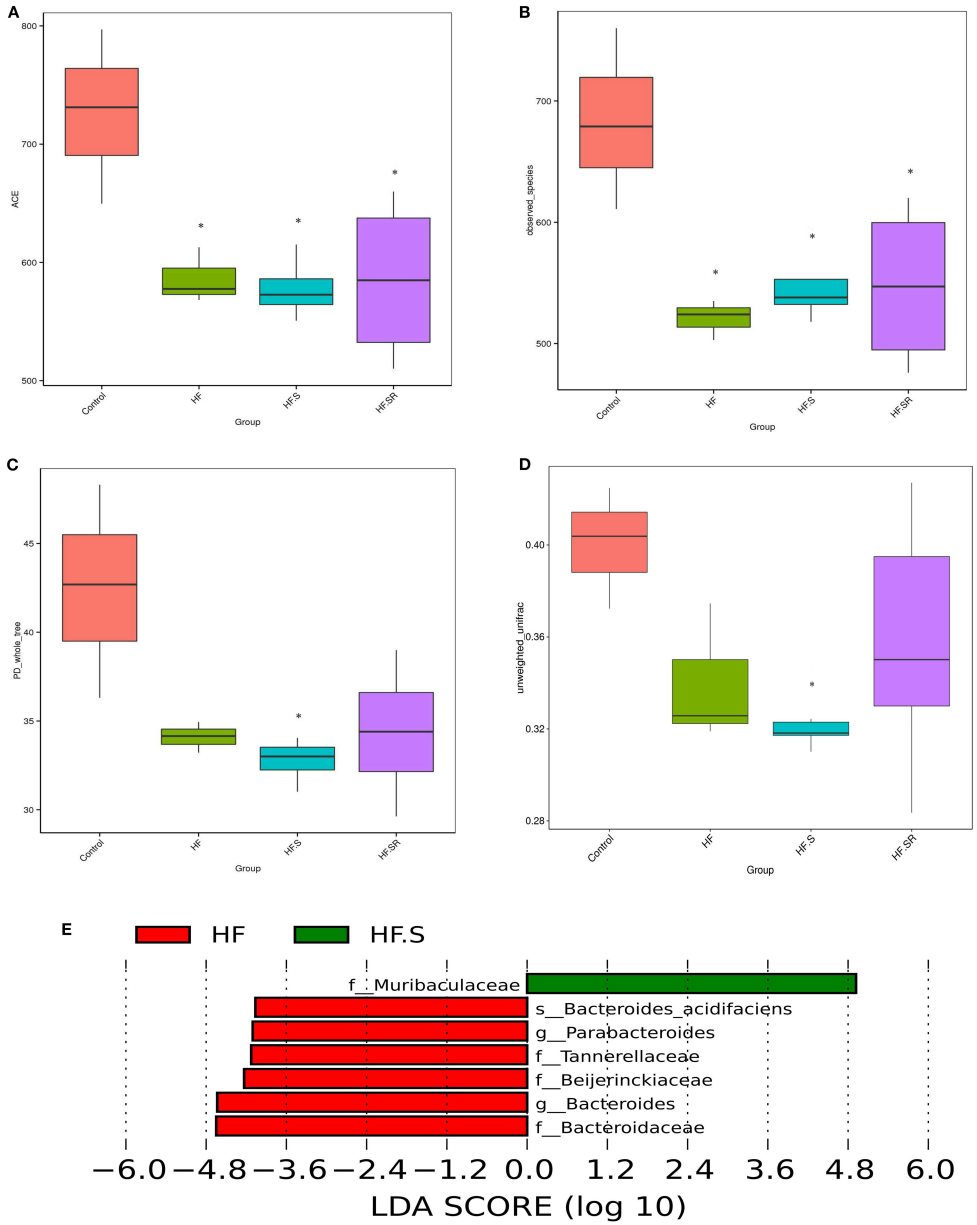
The compositions of the microbial community richness or diversity in the different groups. The community richness was estimated by ACE **(A)** and the observed-species **(B)**. The community diversity is represented by the PD-whole tree **(C)**. Unweighted UniFrac distances revealed significant differences between the controls (*n* = 4) and HF+S mice (*n* = 8) **(D)**. The relative abundance of 6 taxa decreased, while only one increased in the HF+S mice compared with the HF mice (*n* = 5) **(E)**. *P < 0.05 vs. controls.

The compositions of the microbial community richness or diversity were assessed by ACE, observed-species and PD-whole tree. The ACE and observed-species index were significantly higher in the controls than in the HF, HF+S or HF+SR groups (Figures 7A,B, all *P* < 0.05). The PD-whole tree index was lower only in the HF+S group than in the controls (Figure 7C, *P* = 0.023). Moreover, a significant difference in unweighted UniFrac distances was observed between these two groups (*P* = 0.038; Figure 7D).

LefSe analysis showed that at the family and genus levels, the HF+S mice had an increased abundance of *Muribaculaceae*, whereas increased abundances of *Tannerellaceae, Beijerinckiaceae, Bacteroidaceae, Parabacteroides* and *Bacteroides* were found in the HF mice (Figure 7E). When compared to the HF mice, the HF+SR mice possessed more *Muribaculaceae*, but no other difference was found between the two groups.

**Figure 8:**
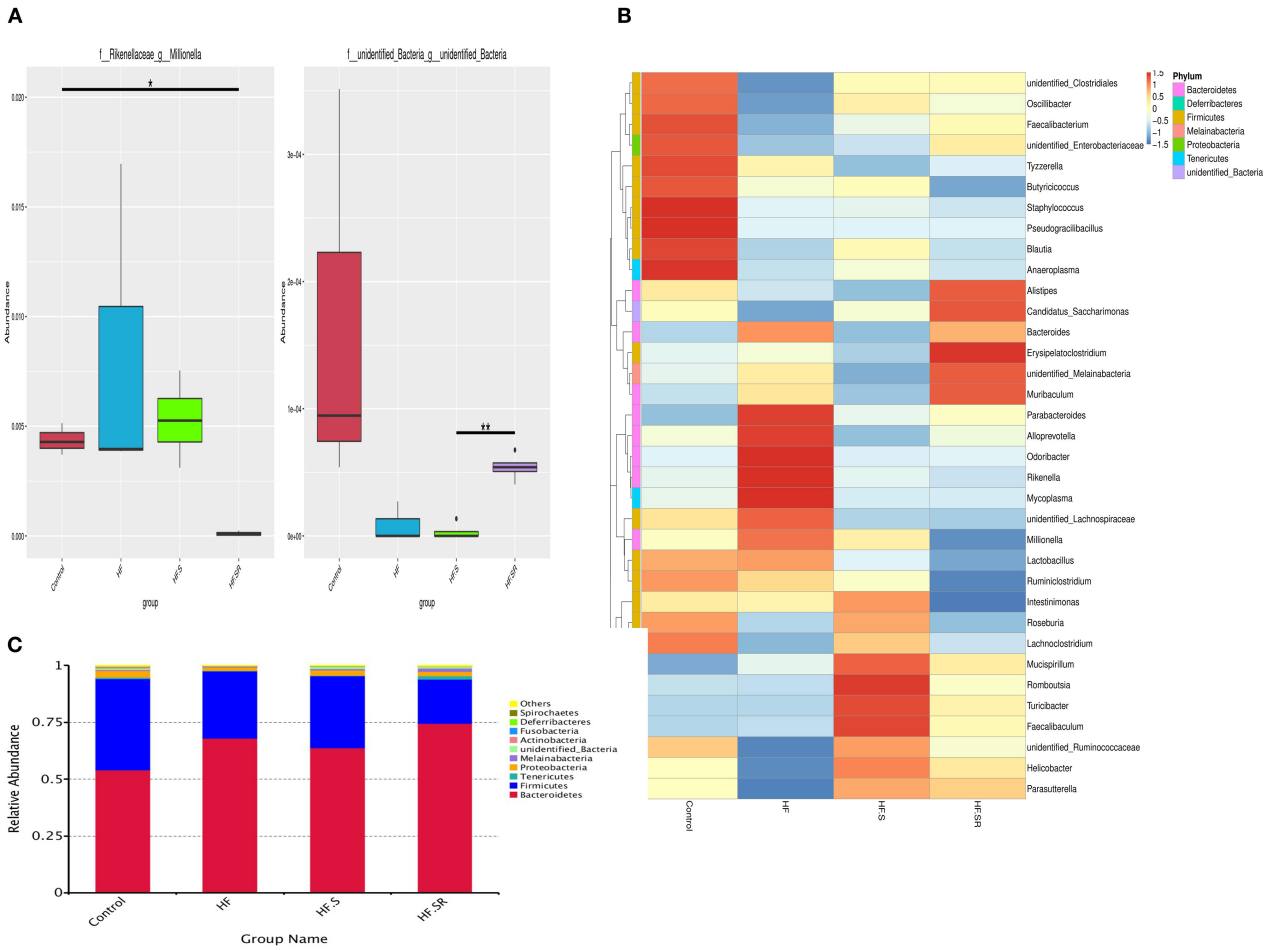
Distribution of bacterial abundance. MetaStat analysis showing the bacterial abundance at the genus level among the control (*n* = 4), HF (*n* = 5), HF+S (*n* = 8) and HF+SR (*n* = 8) mice **(A)**. Top 35 most frequent bacterial genera in the four different groups **(B)**. Relative abundance of the major phyla among the gut microbiota in the four groups. *Bacteroidetes* and *Firmicutes* were the two most abundant bacteria **(C)**. *P < 0.05 vs. controls. **P < 0.05 vs. HF.S group.

MetaStat analysis showed that the spermidine antagonist reduced the relative abundance of *Millionella massiliensis* and other unidentified bacteria (both *P* < 0.05, Figure 8A).

By comparison with the top 35 most frequent bacterial genera, the HF mice had 5 abundant genera (red color) within *Bacteroidetes* and 4 rare genera (blue color) within *Firmicutes*, and the HF+S mice possessed 3 abundant genera within *Firmicutes* and 4 rare genera within *Bacteroidetes*, whereas the HF+SR mice had 2 abundant genera within *Bacteroidetes* and 4 rare genera within *Firmicutes* (Figure 8B).

As shown in Figure 8C, all tested mice dominated two major microbiota among the top 10 phyla: *Bacteroidetes* (54–74%) and *Firmicutes* (19–40%). However, the *Firmicutes*/*Bacteroidetes* ratio was different. The HF+SR mice had more *Bacteroidetes* and less *Firmicutes*.

## Discussion

In the present study, the HF+SR mice presented worsening echocardiographic parameters including LVDs, LVDd, LVVs, and LVVd compared with the HF mice. LVDd and LVVd were better in the HF+S mice than in the HF+SR mice. The mRNA and protein expression levels of galectin-3 in the heart were significantly higher in the HF+SR mice than in the HF mice. In addition, the amount of cardiomyocyte apoptosis was the highest in the mice treated with an antagonist of spermidine. Analysis of the gut microbiota showed that the alpha diversity was significantly higher in the controls than in the HF, HF+S, or HF+SR groups. Moreover, the microbial community diversity decreased significantly and the microbial composition changed considerably after administration of the spermidine antagonist, especially decrease of *Millionella massiliensis*, the *Firmicutes*/*Bacteroidetes* ratio and the increase of *Muribaculaceae*. These findings showed that inhibiting spermidine deteriorated cardiac function and that increasing spermidine was beneficial to cardiac function in HF, and the regulation of the gut microbiota might be a contributing factor to the effect of spermidine on cardiac function.

Dietary spermidine protects against cardiovascular aging (23). In rats with hypertension-induced congestive heart failure, spermidine feeding decreased their systolic blood pressure and prevented cardiac hypertrophy, thus delaying the progression to HF. In humans, high levels of dietary spermidine increase survival and are inversely related to the incidence of cardiovascular disease (13, 14). These results implied that supplementation with spermidine might prevent the occurrence of cardiovascular diseases including HF. However, how was HF affected when changing the levels of spermidine? Our results showed that in HF, reducing spermidine levels by administrating its antagonist led to worsening of the echocardiographic parameters and an increase of galectin-3, a marker of cardiac fibrosis (24), suggesting that decreasing spermidine levels can aggravate cardiac fibrosis and deteriorate cardiac function. Thus, spermidine may be helpful in alleviating cardiac fibrosis during the process of HF and may be beneficial for the recovery of cardiac function. Similar to other potential novel biomarkers for myocardial fibrosis such as secreted frizzled-related protein (25, 26), spermidine may have potential preventive or therapeutic value in myocardial fibrosis and HF.

Spermidine plays many important roles in many aspects of cardiovascular pathophysiology. It can reverse arterial stiffness and restore arterial endothelial function in old mice (27). It reduces lipid accumulation and the formation of atherosclerotic plaques (28, 29). These results show that spermidine has a protective role to arteries and is beneficial for supplying cardiocytes with oxygen and nutrients and maintaining cardiac function. Therefore, maintenance of the appropriate spermidine levels may be important for maintaining cardiac function.

Our results showed that a decrease in spermidine reduced cardiac function, and supplementation with spermidine had a beneficial effect on cardiac function. Although the galectin-3 mRNA levels increased after spermidine supplementation, an increase in protein levels was not observed. Post-transcriptional regulation and translation of mRNA proteins may be responsible for the discrepancy in mRNA and protein levels in galectin-3. The method and duration of spermidine supplementation may be one cause of the small beneficial effect of spermidine supplementation in this study, because the absorption rate may be different when spermidine is administered by injection or orally. In addition, spermidine is a two-edged sword, and only appropriate concentrations can improve cardiac function because inappropriate spermidine levels damage tissue and organs. In rats, spermidine had a potential antipsychotic effect at a low dose (10 mg/kg), but adverse effects appeared at a higher dose (20 mg/kg) (30). It has been reported that excessive intake of spermidine is not without risk. Spermidine showed dose-dependent cytotoxicity in the cultured cells via necrosis and was found to be toxic when its concentrations were above the maximum at which they have been found in food (31). Cancer patients with higher levels of spermidine have a worsed prognosis suggesting that abundant spermidine likely contributes to enhanced growth rates of cancer cells because it is indispensable for cell growth and tumor progression (7, 32).

Moreover, spermidine is not associated with a protective role in cardiovascular disease. It also has an adverse effect on the cardiovascular system. Patients with higher metabolic scores calculated according to the metabolic profile including spermidine levels had worse New York Heart Association functional classes and an adverse prognosis (33). In addition, because cardiac dysfunction is often accompanied by microcirculation disorders (34), a sufficient supply of the myocardium with oxygen and energy by the microcirculation plays very important roles in maintaining normal cardiac function (35, 36). However, Wierich et al. thought that spermidine had no effect on the quantitative characteristics of capillaries or arterioles, including the capillary volume, surface area and length as well as vascular endothelial growth factor-A expression, suggesting that spermidine has no effect on the quantitative structural characteristics of the microcirculation in the aging heart (37). Therefore, the therapeutic role of spermidine may be dose-dependent and identifying a suitable concentration for the improvement of heart failure is very important.

For gut microbiota analysis, consistent with previous observations (3, 4), our results showed that the bacterial richness decreased when HF occurred, regardless of whether spermidine or its antagonist was administered. Furthermore, spermidine increased the abundance of *Muribaculaceae* and its antagonist significantly decreased the *Firmicutes*/*Bacteroidetes* ratio, a widely used marker of gut dysbiosis, meaning that reduced *Firmicutes*/*Bacteroidetes* ratio was related to deteriorated cardiac function. *Firmicutes* and *Bacteroidetes* are the two dominant phyla in the murine and human intestinal microbiota (38), and the *Firmicutes*/*Bacteroidetes* ratio was associated with many disorders. Elderly individuals have a lower *Firmicutes*/*Bacteroidetes* ratio than adults (39), and the *Firmicutes*/*Bacteroidetes* ratio decreased in isoproterenol-induced acute myocardial ischemia rats and chronic intermittent hypoxia exposed pigs (40, 41).

However, different results were also observed in other studies. Lataro et al. thought that the *Firmicutes*/*Bacteroidetes* ratio was not altered in HF model rats subjected to myocardial infarction (42). However, Marques et al. found that a high fiber diet led to a decrease in the *Firmicutes*/*Bacteroidetes* ratio and prevented the development of hypertension and heart failure in hypertensive mice (43). An increased *Firmicutes*/*Bacteroidetes* ratio was also observed in hypertensive patients and rats (44). Perhaps the age of the host, the type of disease, the dietary composition, the environment and other factors can all affect the abundance and composition of gut microbiota (45, 46). The gut microbiota is significantly associated with some polyamines including spermidine. In addition to synthesizing spermidine (14), some gut microorganisms, including *Firmicute* species, also contain spermidine synthase and some accumulate spermidine as the sole polyamine (15). Therefore, the levels and function of spermidine and the microbial community richness or diversity can affect each other. Moreover, decreasing spermidine resulted in the deterioration of HF and a reduction in the *Firmicutes*/*Bacteroidetes* ratio. Thus, spermidine may also play beneficial roles by optimizing the gut microbiota composition.

Several limitations should be discussed. First, in this study, the timing of the administration of spermidine or its antagonist was only 1 week, which was shorter than other reports in which spermidine was administered for 6–12 weeks or longer (13, 14). It is possible that a significant beneficial effect of spermidine in improving cardiac function might be seen if it was administered for a longer period of time. Second, to avoid the effects of sex differences on the roles of spermidine, only female mice were used in this study. The roles of spermidine in cardiac function in male mice need to be investigated further. Third, we did not investigate the appropriate concentrations of spermidine for improving cardiac function and did not measure the variation of spermidine in the blood. Last, our results showed that the abundance of *Millionella massiliensis* decreased significantly in HF mice treated with 4-MCHA, a spermidine synthase inhibitor. However, spermidine can be synthesized by some gut microorganisms. We did not investigate whether *Millionella massiliensis* possessed much greater spermidine synthesis ability than other microbiota and 4-MCHA acted as a spermidine synthase inhibitor by decreasing the abundance of *Millionella massiliensis*.

In summary, these findings showed that inhibiting spermidine by inhibiting its synthesis deteriorated cardiac function, while increasing spermidine improved cardiac function, and the regulation of gut microbiota and cardiac fibrosis might be a factor in the effects of spermidine on the modulation of cardiac function.

## Data Availability Statement

The original contributions presented in the study are included in the article/Supplementary Material, further inquiries can be directed to the corresponding author.

## Ethics Statement

The animal study was reviewed and approved by Institutional Ethics Committee of Guangzhou First People's Hospital.

## Author Contributions

PC designed the study. Material preparation and data collection were performed by ZG, YC, ZL, and SL. ZG and YC conducted the statistical analyses. PC, ZG, and YC drafted the paper, which was reviewed by all authors. All authors read and approved the final manuscript.

## Funding

This work was supported by Natural Science Foundation of Guangdong Province (Grant Number 2020A1515010384), the Science and Technology Planning Project Foundation of Guangzhou (Grant Number 201804010463), and National Natural Science Foundation of China (Grant Number 81770398).

## Conflict of Interest

The authors declare that the research was conducted in the absence of any commercial or financial relationships that could be construed as a potential conflict of interest.

## Publisher's Note

All claims expressed in this article are solely those of the authors and do not necessarily represent those of their affiliated organizations, or those of the publisher, the editors and the reviewers. Any product that may be evaluated in this article, or claim that may be made by its manufacturer, is not guaranteed or endorsed by the publisher.
