# Supplementary figures and images for: Spermidine Affects Cardiac Function in Heart Failure Mice by Influencing the Gut Microbiota and Cardiac Galectin-3
Source: Front Cardiovasc Med. 2021 Dec 2;8:765591. doi: 10.3389/fcvm.2021.765591 (PMC8674475; doi:10.3389/fcvm.2021.765591)

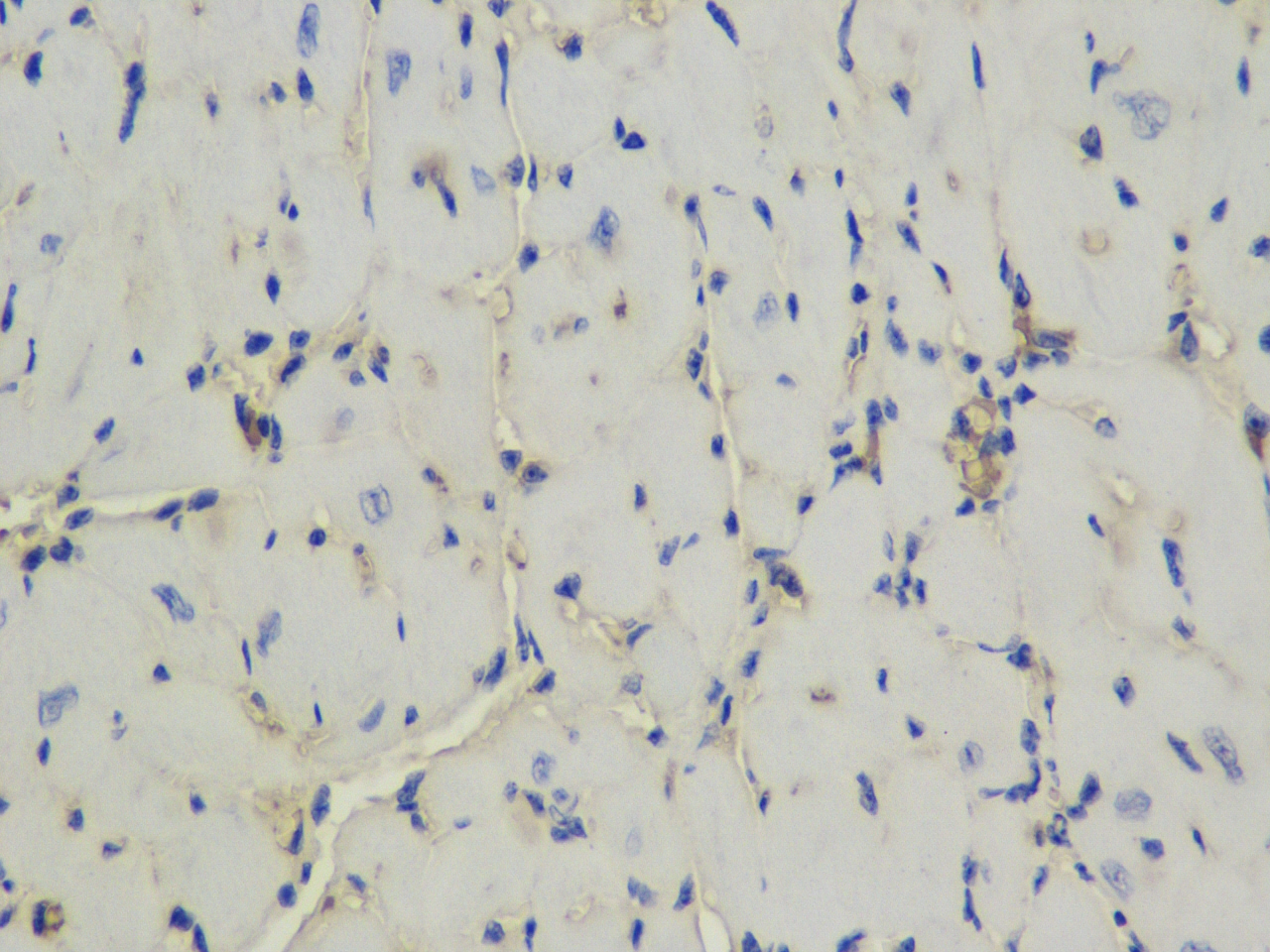

Supplement: Supplementary file 1 [file Data_Sheet_1.zip › additional files/AOD/HF+S.jpg]

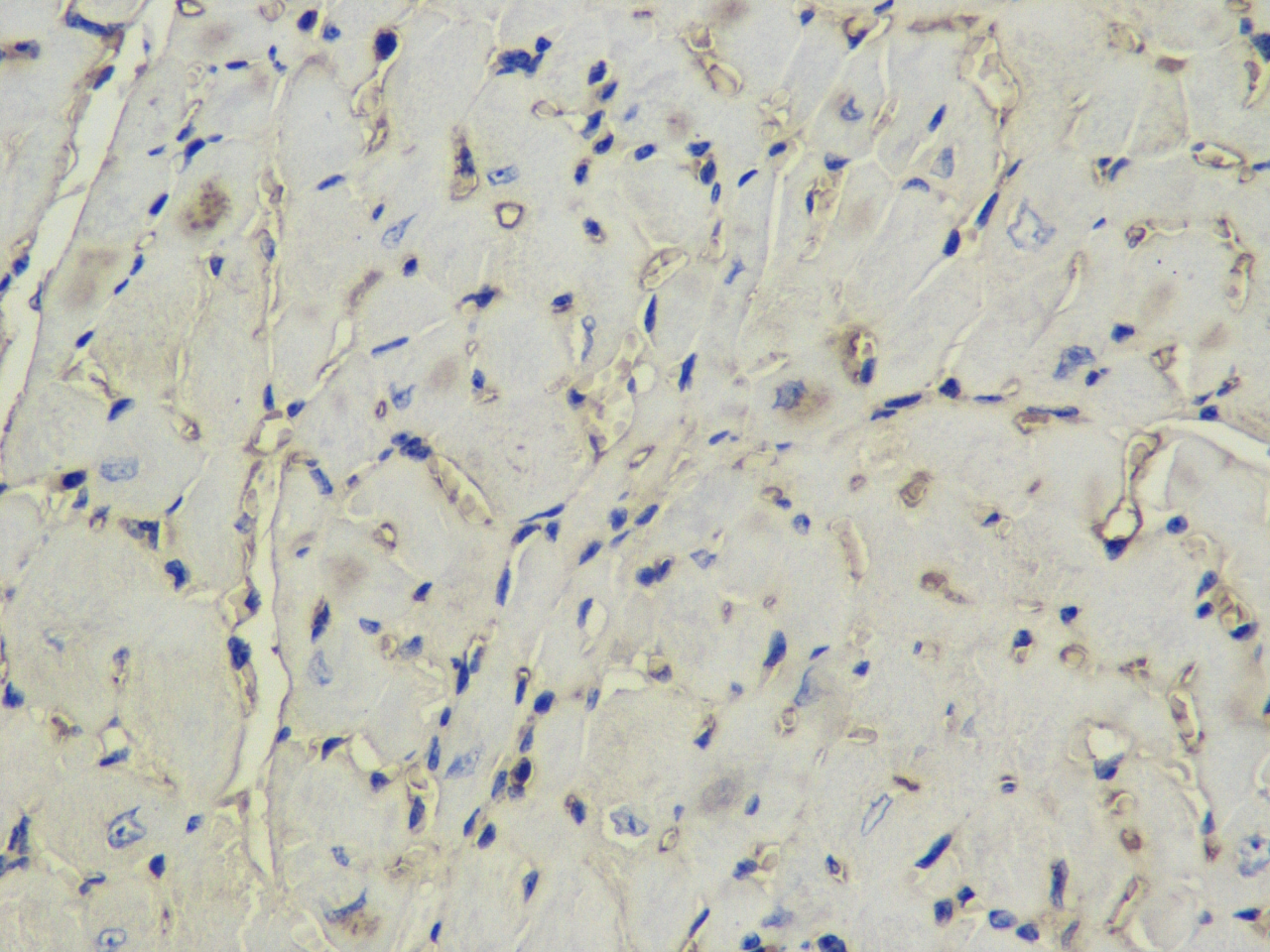

Supplement: Supplementary file 1 [file Data_Sheet_1.zip › additional files/AOD/HF+SR.jpg]

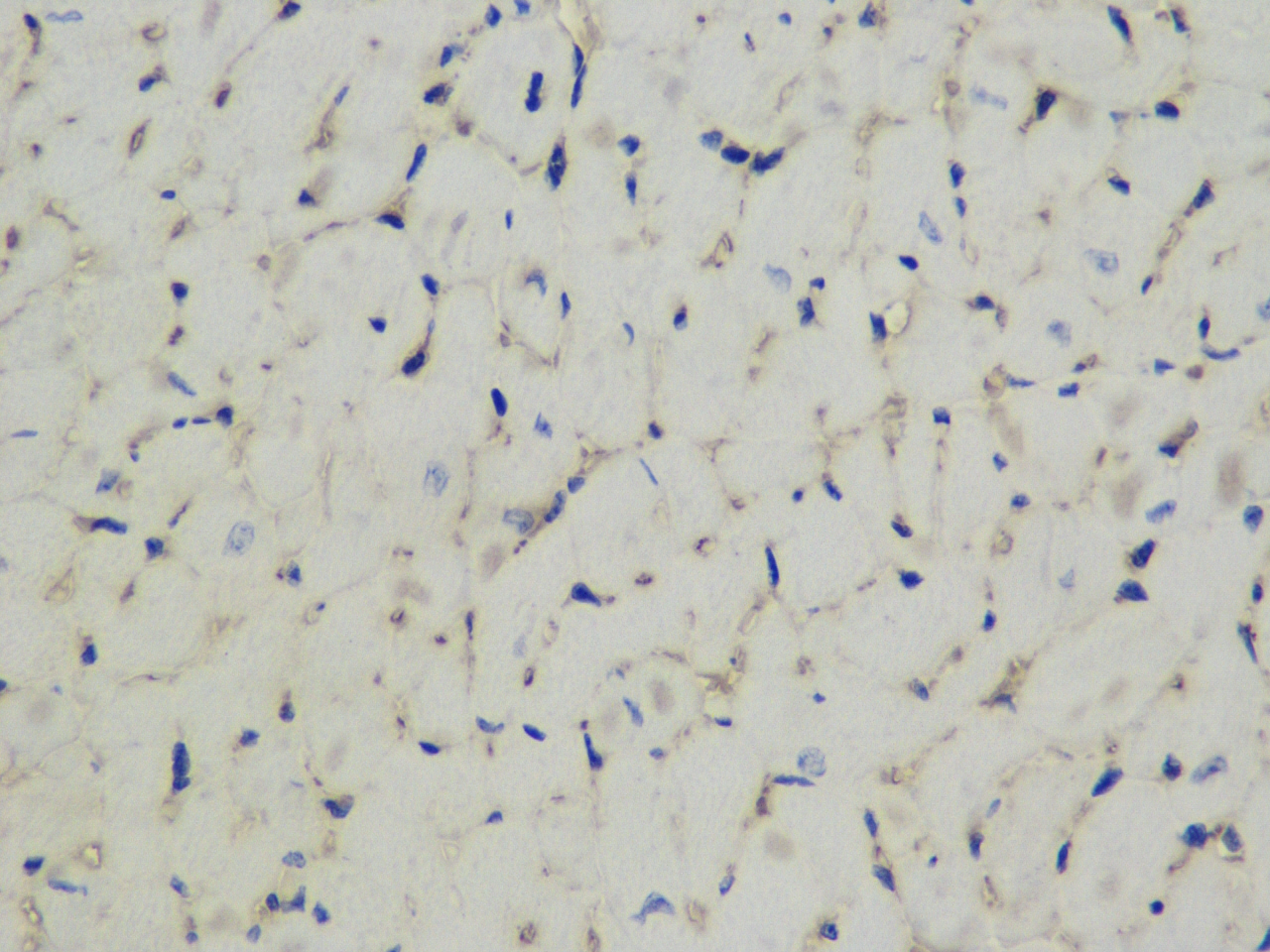

Supplement: Supplementary file 1 [file Data_Sheet_1.zip › additional files/AOD/HF.jpg]

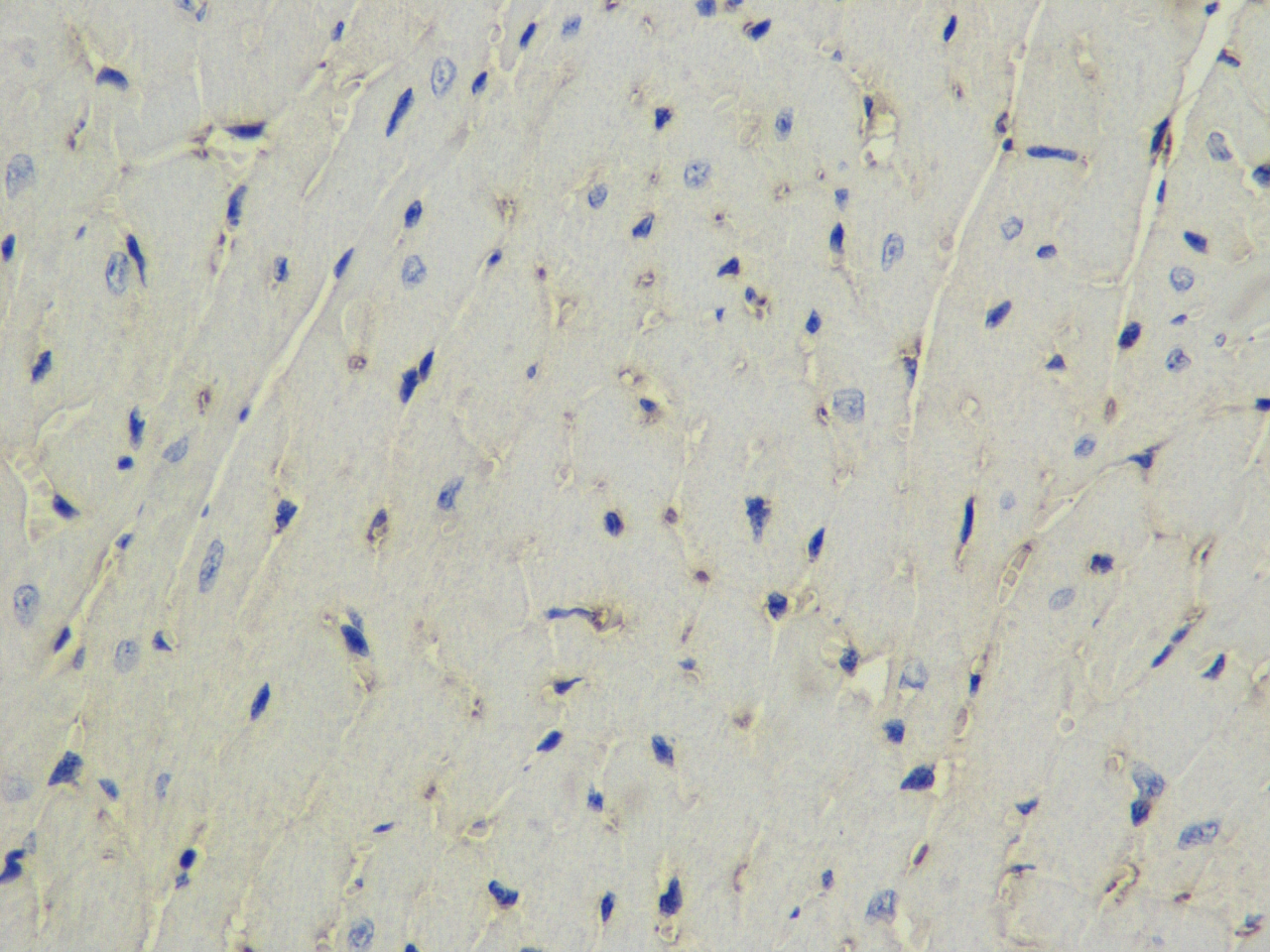

Supplement: Supplementary file 1 [file Data_Sheet_1.zip › additional files/AOD/control.jpg]

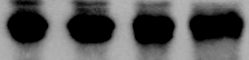

Supplement: Supplementary file 1 [file Data_Sheet_1.zip › additional files/B-actin.jpg]

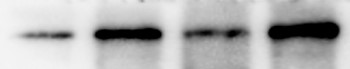

Supplement: Supplementary file 1 [file Data_Sheet_1.zip › additional files/Galctin-3.jpg]

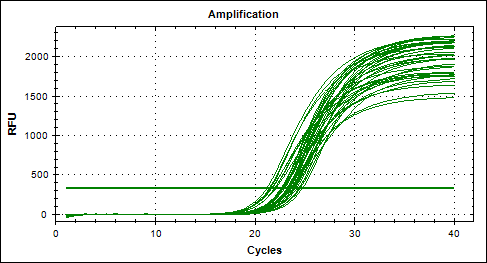


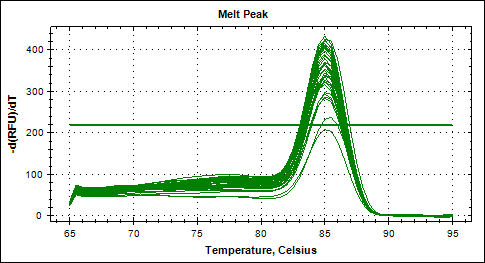

Supplement: Supplementary file 1 [file Data_Sheet_1.zip › additional files/PCR.docx]

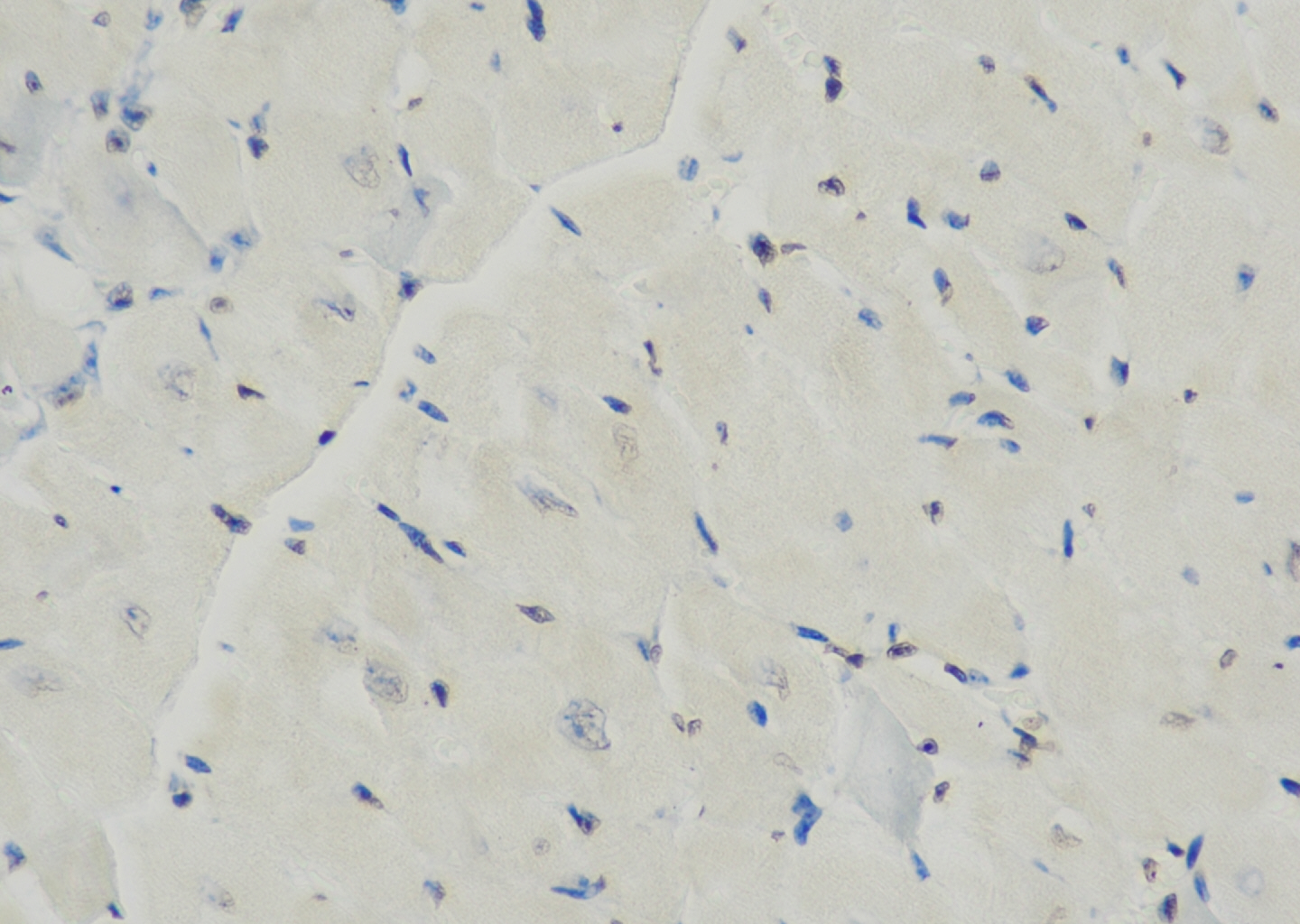

Supplement: Supplementary file 1 [file Data_Sheet_1.zip › additional files/TUNEL/Control.jpg]

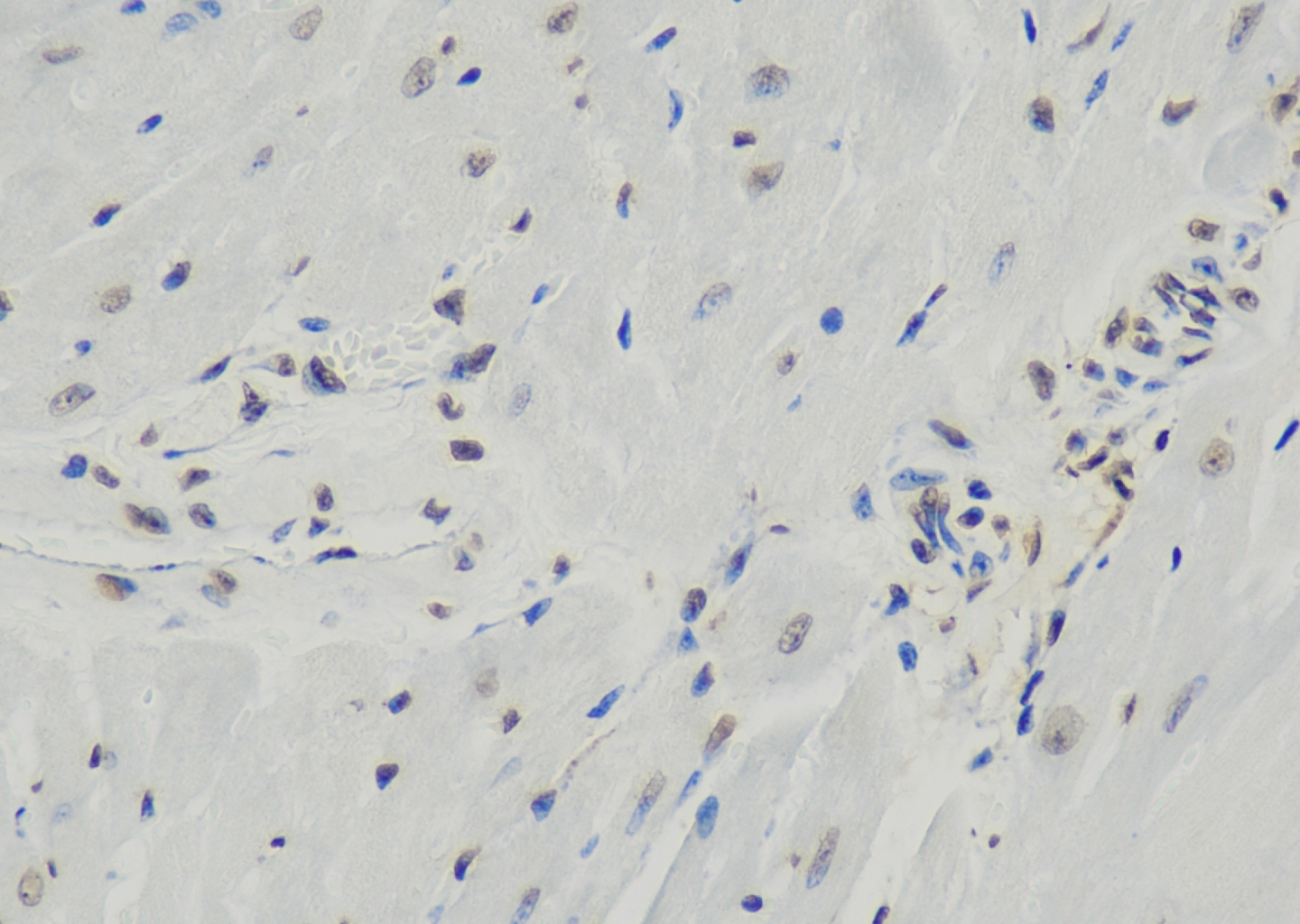

Supplement: Supplementary file 1 [file Data_Sheet_1.zip › additional files/TUNEL/HF+S.jpg]

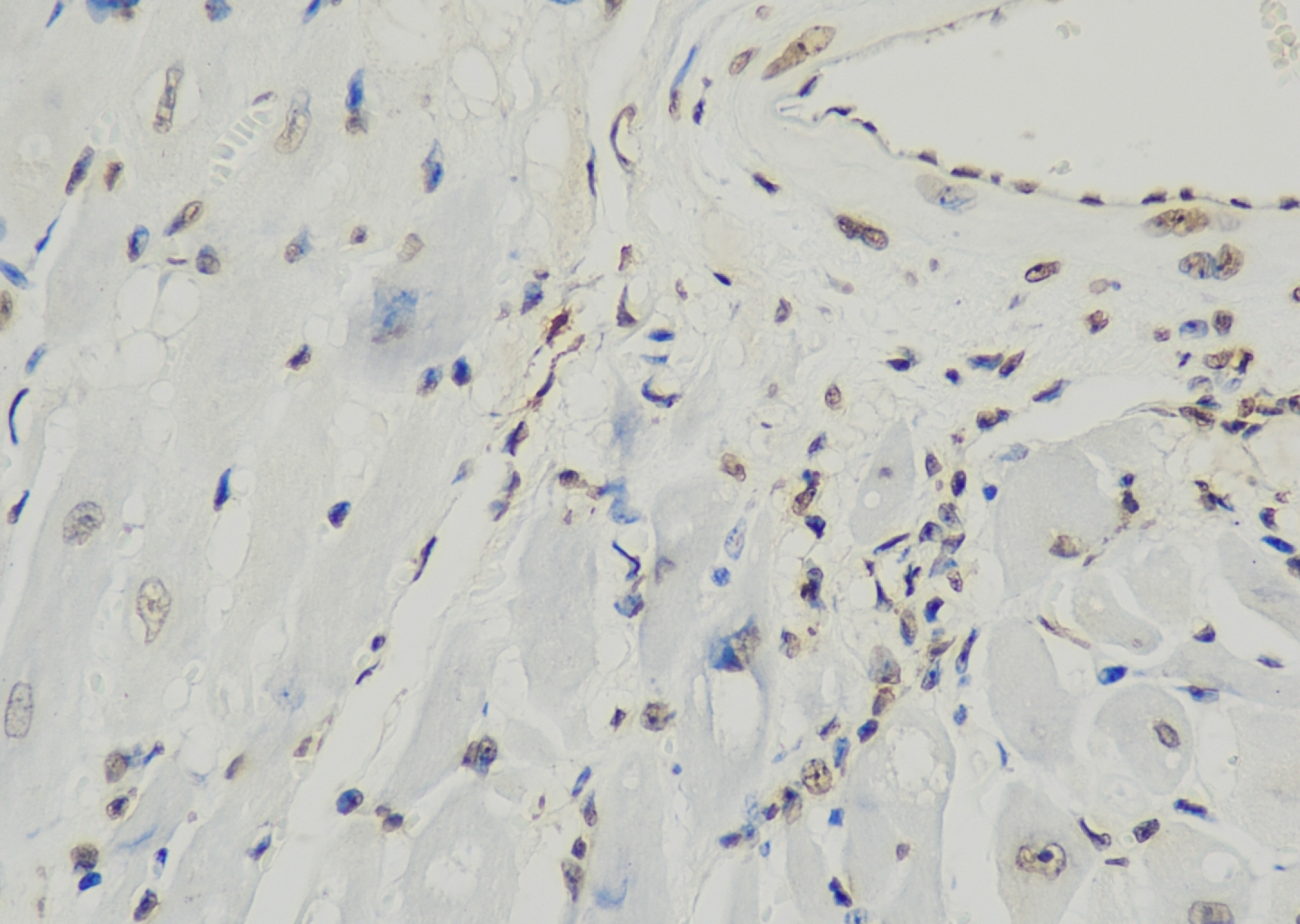

Supplement: Supplementary file 1 [file Data_Sheet_1.zip › additional files/TUNEL/HF+SR.jpg]

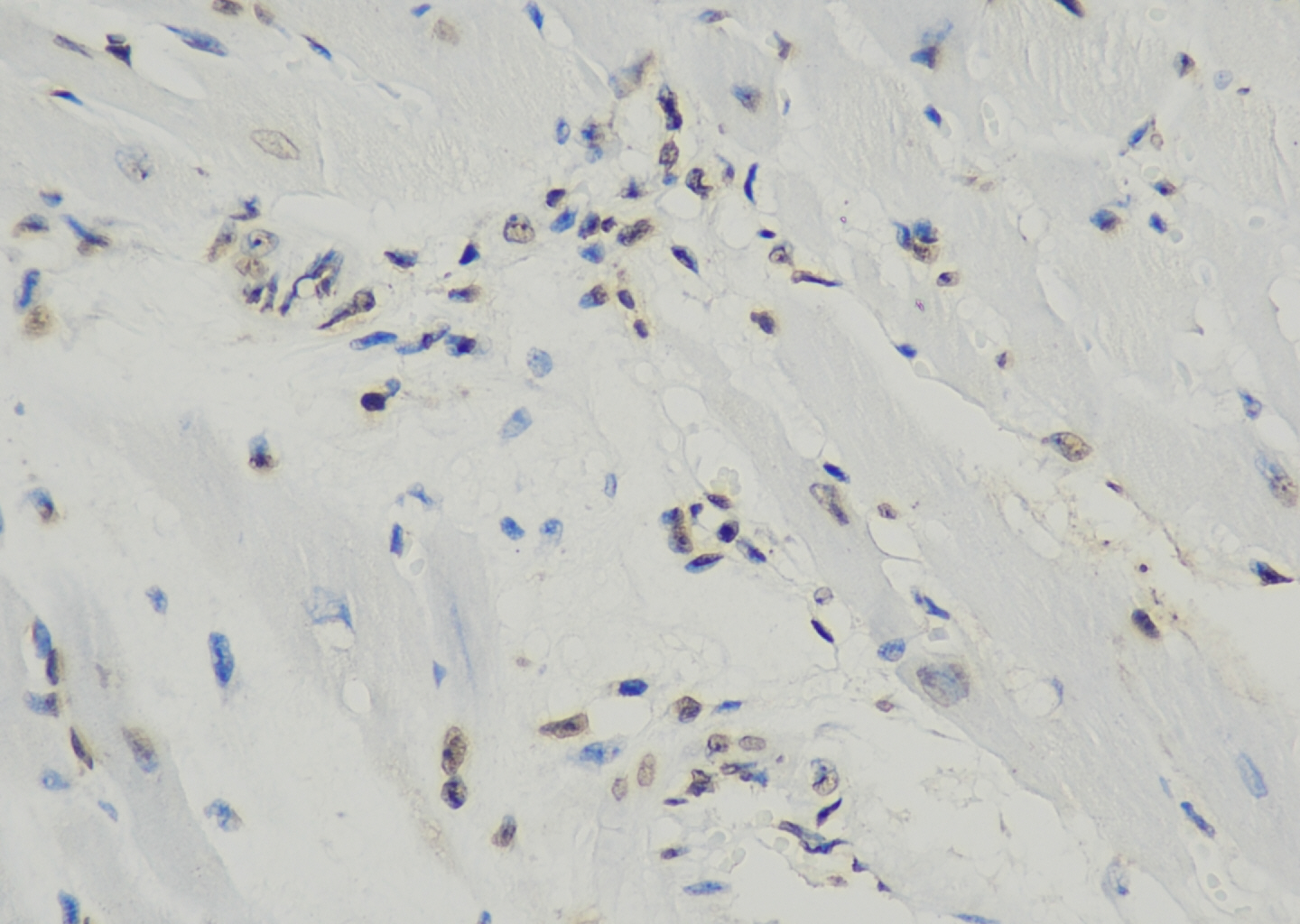

Supplement: Supplementary file 1 [file Data_Sheet_1.zip › additional files/TUNEL/HF.jpg]

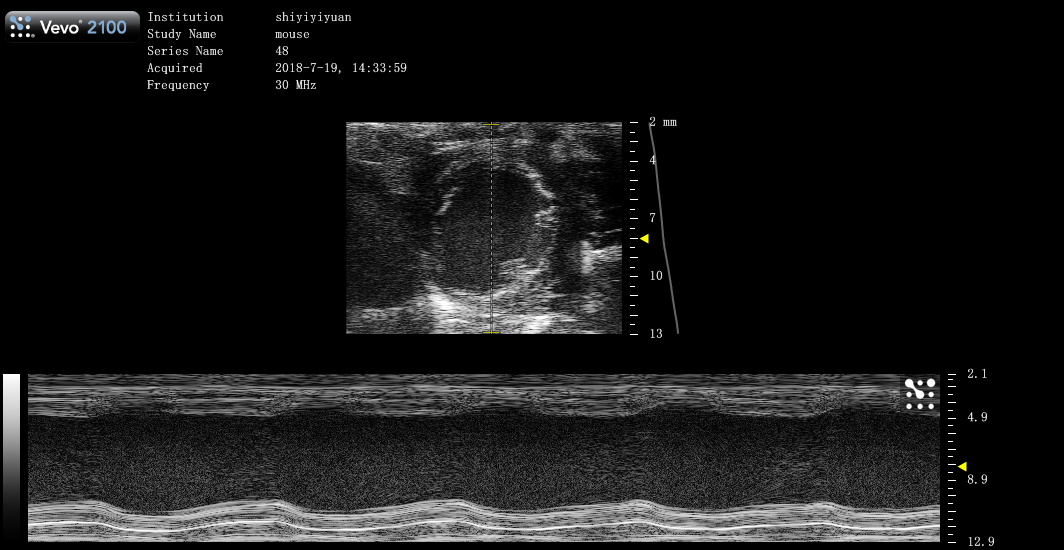

Supplement: Supplementary file 1 [file Data_Sheet_1.zip › additional files/figure 1/2018-07-19HF+SR╢╠╓ß48''.tif]

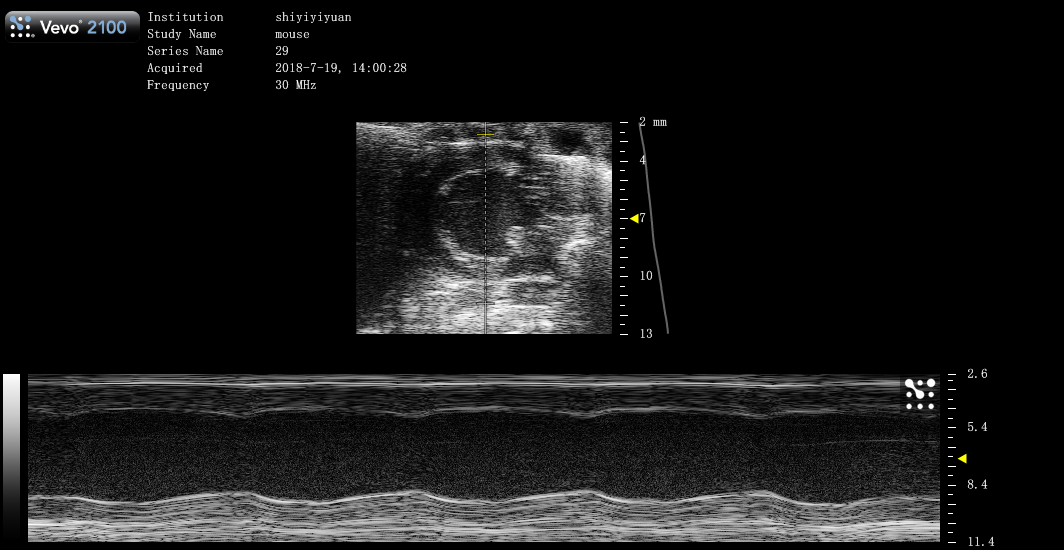

Supplement: Supplementary file 1 [file Data_Sheet_1.zip › additional files/figure 1/2018-07-19HF+S╢╠╓ß29''.tif]

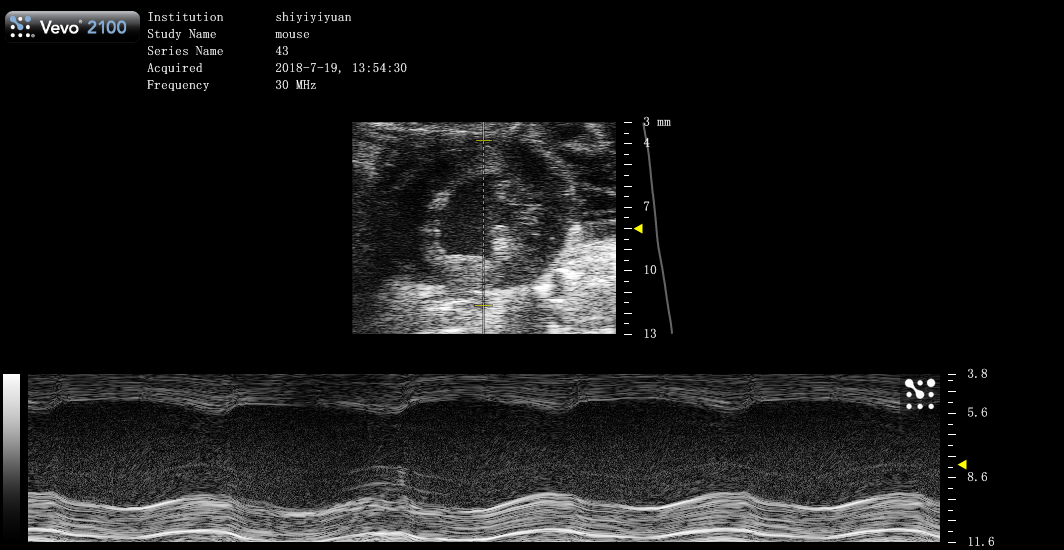

Supplement: Supplementary file 1 [file Data_Sheet_1.zip › additional files/figure 1/2018-07-19HF╢╠╓ß43''.tif]

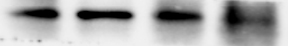

Supplement: Supplementary file 1 [file Data_Sheet_1.zip › additional files/spermidine.jpg]

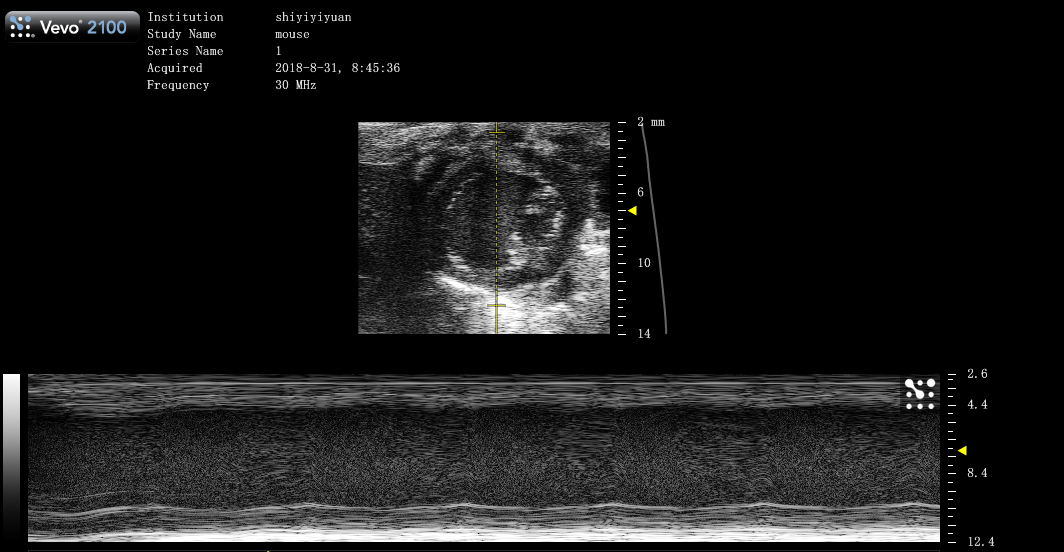

Supplement: Supplementary file 1 [file Data_Sheet_1.zip › additional files/‏figure 2/2018-08-31HF+SR短轴1''.tif]

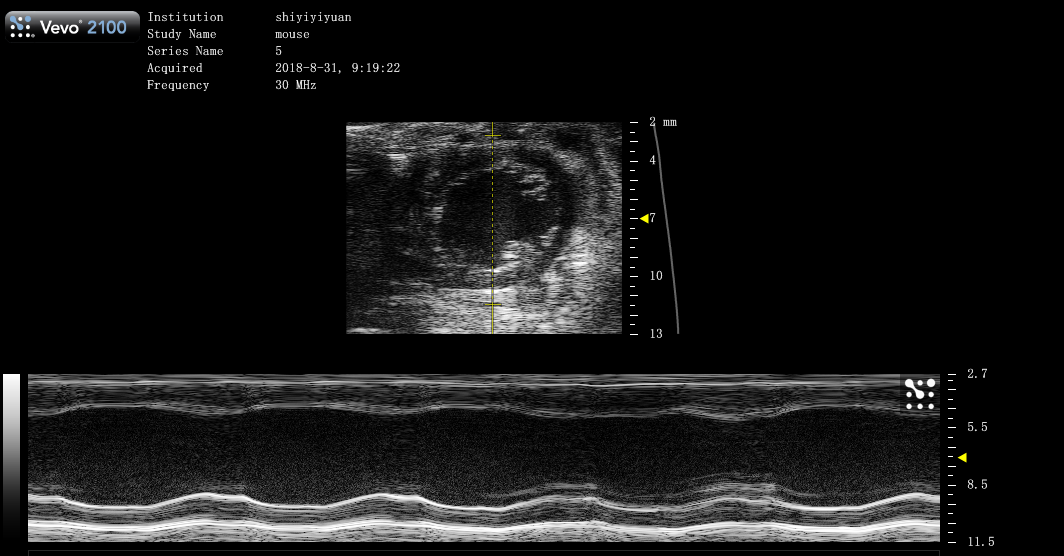

Supplement: Supplementary file 1 [file Data_Sheet_1.zip › additional files/‏figure 2/2018-08-31HF+S短轴5''.tif]

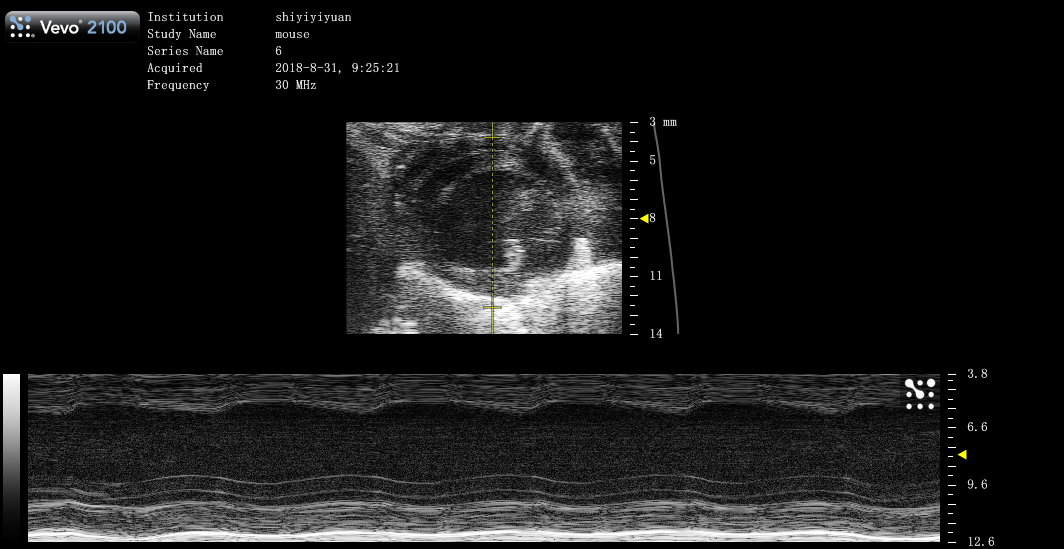

Supplement: Supplementary file 1 [file Data_Sheet_1.zip › additional files/‏figure 2/2018-08-31HF短轴6''.tif]

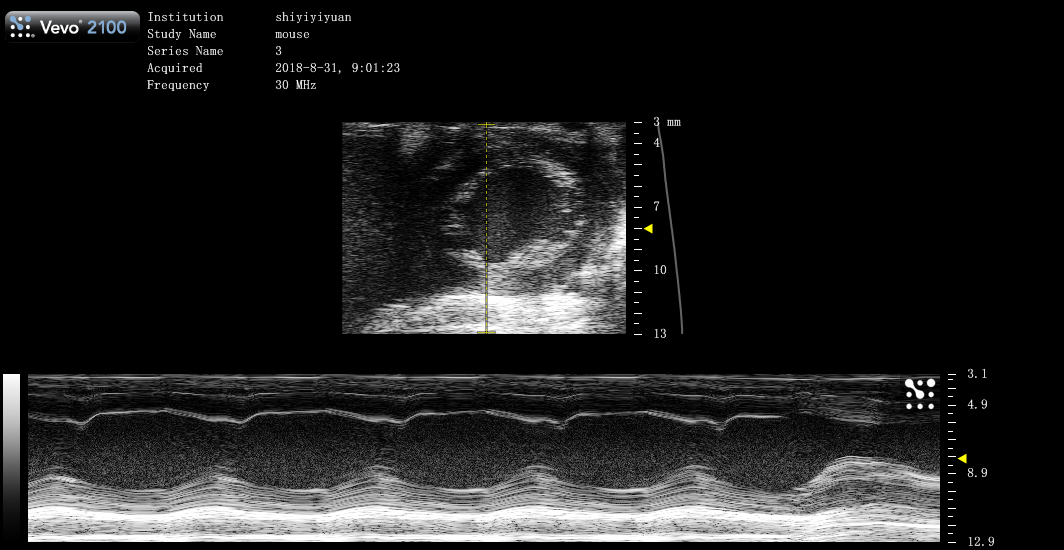

Supplement: Supplementary file 1 [file Data_Sheet_1.zip › additional files/‏figure 2/2018-08-31control短轴3''.tif]
